# Transcriptome analysis of responses in *Brachypodium distachyon* overexpressing the BdbZIP26 transcription factor

**DOI:** 10.1186/s12870-020-02341-3

**Published:** 2020-04-20

**Authors:** Ruth C. Martin, Brent A. Kronmiller, James E. Dombrowski

**Affiliations:** 1 United States Department of Agriculture, Agricultural Research Service, National Forage Seed Production Research Center, 3450 SW Campus Way, Corvallis, OR 97331 USA; 2 Center for Genome Research and Biocomputing, Oregon State University, Corvallis, OR 97331 USA

**Keywords:** bZIP26, *Brachypodium*, Salt stress, RNA-Seq, Transcriptome

## Abstract

**Background:**

Biotic and abiotic stresses are the major cause of reduced growth, persistence, and yield in agriculture. Over the past decade, RNA-Sequencing and the use of transgenics with altered expression of stress related genes have been utilized to gain a better understanding of the molecular mechanisms leading to salt tolerance in a variety of species. Identification of transcription factors that, when overexpressed in plants, improve multiple stress tolerance may be valuable for crop improvement, but sometimes overexpression leads to deleterious effects during normal plant growth.

**Results:**

*Brachypodium* constitutively expressing the BdbZIP26:GFP gene showed reduced stature compared to wild type plants (WT). RNA-Seq analysis comparing WT and bZIP26 transgenic plants revealed 7772 differentially expressed genes (DEGs). Of these DEGs, 987 of the DEGs were differentially expressed in all three transgenic lines. Many of these DEGs are similar to those often observed in response to abiotic and biotic stress, including signaling proteins such as kinases/phosphatases, calcium/calmodulin related proteins, oxidases/reductases, hormone production and signaling, transcription factors, as well as disease responsive proteins. Interestingly, there were many DEGs associated with protein turnover including ubiquitin-related proteins, F-Box and U-box related proteins, membrane proteins, and ribosomal synthesis proteins. Transgenic and control plants were exposed to salinity stress. Many of the DEGs between the WT and transgenic lines under control conditions were also found to be differentially expressed in WT in response to salinity stress. This suggests that the over-expression of the transcription factor is placing the plant in a state of stress, which may contribute to the plants diminished stature.

**Conclusion:**

The constitutive expression of BdbZIP26:GFP had an overall negative effect on plant growth and resulted in stunted plants compared to WT plants under control conditions, and a similar response to WT plants under salt stress conditions. The results of gene expression analysis suggest that the transgenic plants are in a constant state of stress, and that they are trying to allocate resources to survive.

## Background

Biotic and abiotic stresses cause major losses in agricultural production due to reduced growth, persistence, and yield in crop species. Identification of viable strategies to reduce yield losses and improve stress tolerance is important for the goal of global food security. To develop these strategies, it is important to understand how plants sense and respond to stresses at the molecular level to facilitate the identification of target genes or pathways for improved stress tolerance. Much has been learned over the years concerning key components involved in the perception, signaling and response of plants to various biotic and abiotic stresses. Plants utilize receptors, membrane channels, various types of kinases and phosphatases, ion fluxes, calcium/calmodulin interactive proteins, reactive oxygen species (ROS) and phytohormones for sensing and signaling different types of stresses [1]. Transcription factors (TFs) participate in the signaling cascades, and are a key component in regulating gene expression in response to biotic and abiotic stresses and in growth and development [2, 3]. While there are about 60 different transcription factor families in plants [4], several families are more frequently associated with biotic and abiotic stress responses in plants, such as MYB (v-myb avian myeloblastosis viral oncogene homolog) [5], bHLH [6], AP2/ERF [7, 8], WRKY [9], NAC [10], and bZIP transcription factors [11–13].

Over the last several years, the transcriptomes of various plant species have been mined for the presence of basic leucine zipper (bZIP) transcription factors [11–14]. The bZIP family of transcription factors is characterized by having a basic region that binds DNA and a leucine zipper dimerization motif (N - X_7_ - R/K - X_9_ - L - X_6_ – L - X_6_ – L) [11]. This is one of the larger groups of plant transcription factors, with members involved in many aspects of plant growth and development, such as germination, seed maturation, senescence, and flowering; but also with members involved in biotic and abiotic stress responsive networks [11]. To gain a better understanding of the role of bZIP transcription factors during stress responses, several members of this family have been overexpressed or silenced in different plant species to assess their impact on abiotic/biotic stress tolerance and their effect on plant growth and development. Wheat lip19 bZIP transcription factor (*Wlip19*), which was activated by low temperature, drought and ABA, conferred improved abiotic stress tolerance when overexpressed in tobacco [15]. Overexpression of a *Brachypodium* bZIP10 transcription factor (Bradi1g30140.1) in *Brachypodium* induced expression of several protective oxidative stress genes, leading to increased oxidative stress tolerance [16], with minimal effects on plant growth and development. A wheat *TabZIP60* gene, which was strongly induced by polyethylene glycol, salt, cold and abscisic acid (ABA) treatments, was able to improve drought, salt and freezing stress tolerance when overexpressed in *Arabidopsis*, with the seedlings displaying increased sensitivity to ABA [17]. Another wheat transcription factor, TabZIP174, was shown to be localized to the nucleus and was found to be responsive to ABA. Transgenic *Arabidopsis* overexpressing *TabZIP174* had better drought tolerance with higher proline, soluble sugar, and leaf chlorophyll contents [18]. The drought-, ROS-, and ABA-inducible rice bZIP62 transcription factor, when over-expressed in rice using an enhanced promoter (35S plus a VP64 universal transcription activation module) displayed increased drought and oxidative stress tolerance [19]. When a heat-, salinity-, cold- and dehydration-responsive wheat bZIP transcription factor was overexpressed in *Arabidopsis*, the transgenic plants were more tolerant to salinity, drought, heat and oxidative stress, and had increased levels of ROS scavenging enzymes and decreased levels of ROS [14]. It is clear that the bZIP family of transcription factors have an integral role in the response to abiotic stresses in plants.

Recently, ninety-six bZIP transcription factors in *Brachypodium distachyon* were characterized and profiled for their expression levels in various plant tissues, and in their response to a range of environmental stresses, heavy metal stresses, and phytohormones [12]. During their studies, they found that bZIP27 (Bradi1g76690; Grassius bZIP26, our bZIP26) was up-regulated in response to cold, heat, polyethylene glycol, sodium chloride, salicylic acid, benzylaminopurine and abscisic acid. Additionally, expression of bZIP27 was down-regulated with exposure to heavy metals, such as manganese, cadmium and lead. They also predicted that bZIP27 was capable of homo- or hetero-dimerization with other bZIP transcription factors. The nearest homologs of Bradi1g76690 in rice are Os03g03550 (OsbZIP25), Os08g43090 (OsbZIP 68) and Os10g38820 (OsbZIP78); and the closest homologues in Arabidopsis are At4g38900 (AtbZIP29) and At2g21230 (AtbZIP30) [12]. A BLAST at NCBI with the protein sequence encoded by Bradi1g76690 indicated homology to RF2a and RF2b proteins. Members of this family were originally identified as proteins interacting with the Box II sequences in the promoter of rice tungro virus, and are responsible for activating the transcription of the virus [20, 21]. This manuscript describes the effects of overexpressing bZIP26:GFP under the control of a constitutive promoter on plant growth, and the analysis of transcriptome differences between wildtype *Brachypodium* (WT) and transgenic *Brachypodium* plants (TR) under control and long-term salt stress conditions.

## Results

### Transgene expression analysis and phenotype of transgenic lines compared to WT

**Fig. 1:**
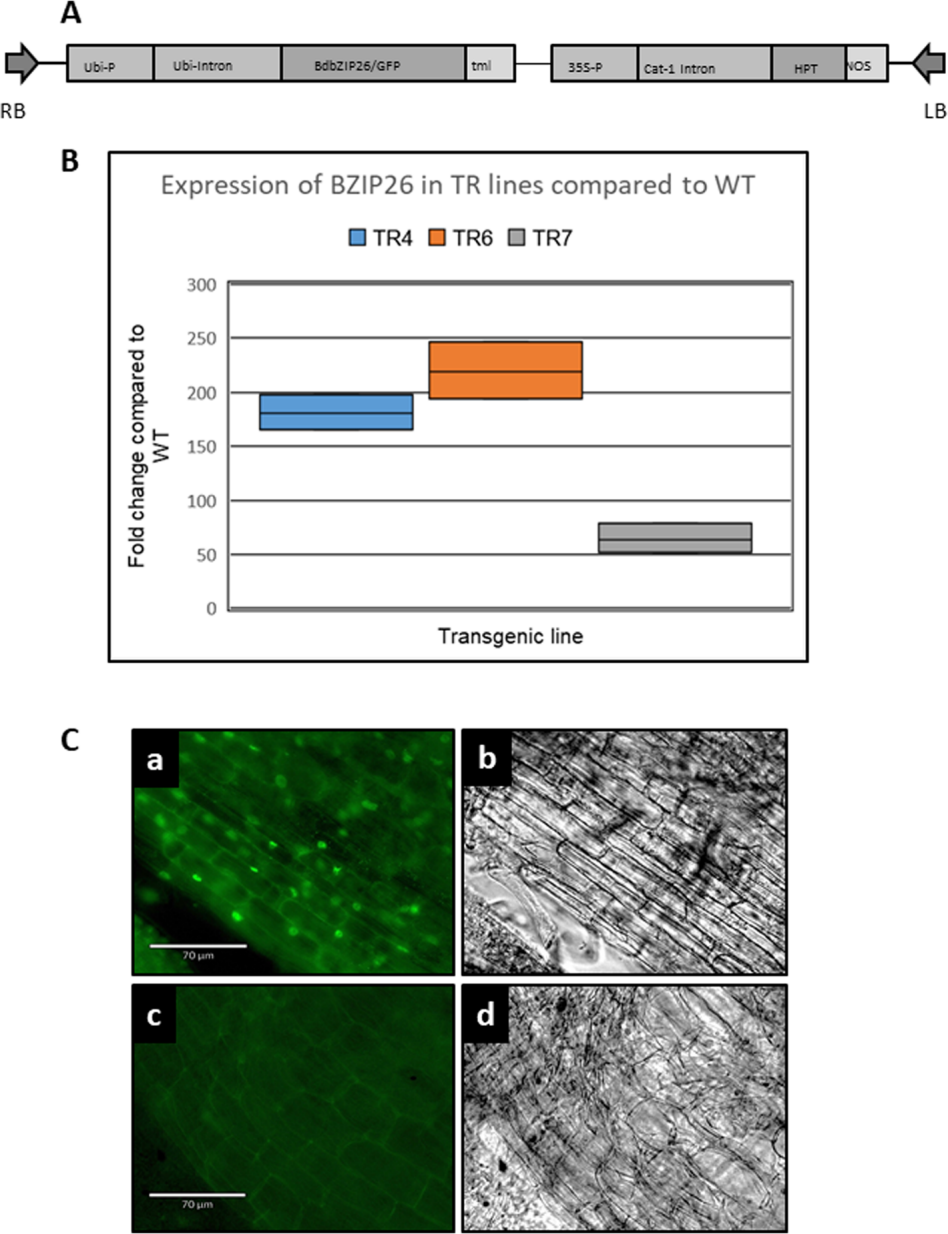
Overexpression of bZIP26:GFP. **a** Vector construct used for bZIP26:GFP transformation. **b** Expression fold level increase of bZIP26 in transgenic lines 4, 6, and 7 (TR4, TR6, TR7) compared to wild type plants. **c** bZIP26:GFP localization, a) Fluorescence micrograph of bZIP26:GFP localization in root tissue and, b) Bright light image of same root tissue; c) Fluorescence micrograph of non-transformed *Brachypodium*, and d) Bright light image of same root tissue. Bar = 70 μM

Plants (TR4, TR6, and TR7) from three independent transformation events were tested for the expression level of the bZIP26 compared to WT plants under control conditions. Transgenic plants showed a range of transgene expression levels compared to WT as shown in Fig. 1B. TR7, with a 64-fold increase compared to WT, had the lowest expression level of the three transgenic lines. TR4 and TR6 had higher fold increases in expression compared to WT, with averages of 181 and 219, respectively. Next, we wanted to determine where the protein was localized within the cell. Young root tissue of transgenic and WT plants was examined with an Echo Revolve D137 microscope using brightfield and fluorescence lighting (Echo Laboratories; San Diego, CA). Images of a transgenic root section are shown in Fig. 1C-a (fluorescent) and C-b (brightfield). The fluorescence signal is strong in the nuclei, which is consistent with its activity as a transcription factor. A slight shadow of fluorescence is visible along the margins of the cells, but this is present in the WT root under fluorescence as well (Fig. 1C-c). The brightfield image of WT root is shown in Fig. 1C-d.

**Fig. 2:**
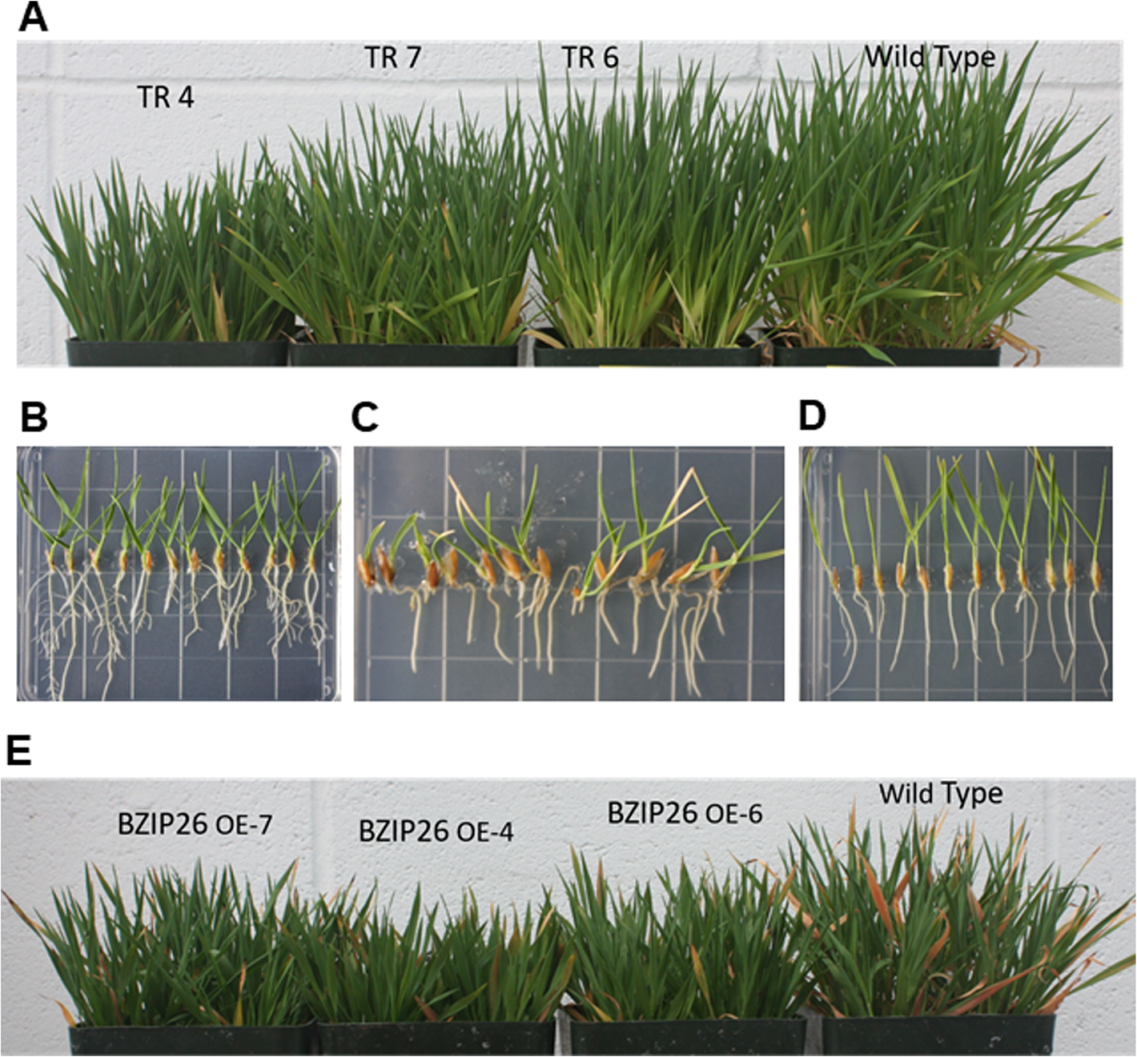
Growth of bZIP26:GFP transgenic lines (TR) compared to wild type (WT) plants. **a** TR4, TR6, TR7 and WT plants grown for 7.5 weeks in a Conviron PGW40 growth chamber. **b-d** Growth of pre-germinated seeds on Murashige and Skoog medium in a Tissue Culture Conviron TC80 chamber. For all plates, seedlings are arranged, from left to right; 3 WT plants, 3 TR4 plants, 3 TR6 plants, and 3 TR 7 plants and plates are set vertical in the growth chamber. Seedlings were grown on the following medium for the time indicated: **b** ½ MS medium with no hormones for 12 d; **c** ½ MS media with 2 μM ABA for 21 d; or **d** ½ MS media with 20 μM GA3 for 7d; **e** Effect of salinity on growth of WT and TR plants, which were grown under the same conditions as **a**, but they were treated with 100 mM NaCl for the last 3 weeks

*Brachypodium* WT and homozygous TR4, TR6 and TR7 plants were evaluated to determine the effect of the transgene on growth under normal and salinity stress conditions. Surprisingly, the untreated transgenic plants were reduced in stature when compared to WT plants (Fig. 2a), with the TR4 plants being the shortest. The analysis of 3-week-old plants revealed changes in internode lengths. The distance between the first two nodes was significantly less in TR4 (12.65+/− 1.1 mm) and TR6 (14.0+/−.89 mm) compared to WT (17.8+/− 1.3 mm) (data not shown; TR7 not measured), however the length of the next internode up was not significantly different between WT and TR lines. The reduced stature was also evident in young seedlings grown on ½ strength MS medium [22], as shown in Fig. 2b. The roots and shoots of the WT plants (seedlings 1–3, starting at the left side of the plate) were more developed than TR4 (seedlings 4–6), TR6 (seedlings 7–9) and TR7 (seedlings 10–12). To determine if hormones could alleviate the differences in growth, we placed seeds on ½ strength MS media containing 2 μM ABA, but on this media, neither WT nor TR lines were able to germinate (data not shown). When seeds were germinated first and then placed on ½ MS with 2 μM ABA, surprisingly the transgenic plants initially grew faster than the WT plants with longer roots and shoots, but then began to show necrosis at the tips of their leaves after 2 weeks of growth (Fig. 2c). To determine if the dwarf phenotype of the transgenic plants could be rescued with GA, seeds were germinated and then put on ½ MS medium containing 20 μM GA3. The transgenic plants grew similar to, or slightly faster than the WT plants on GA-supplemented media (Fig. 2d). We also wanted to see how the transgenic plants would perform under salinity stress. For this experiment, 4–5-week-old plants were treated with 100 mM salt for 3 weeks. Exposure to salt stress caused stunting in both WT and transgenic plants (Fig. 2e). The WT plants were still larger than the transgenic plants, but the difference was not as pronounced as that seen under control conditions (Fig. 2a), and the WT plants exhibited more necrosis at the tips of their leaves (Fig. 2e).

### RNA-sequencing

To investigate potential molecular mechanisms associated with the dwarf phenotype in the transgenic plants, and the effects of salinity stress in WT and transgenic plants, RNA-Seq of WT, TR4, TR6, and TR7 plants under control and long term salt stress (100 mM salt for 3 wks) was performed. Sequencing produced libraries averaging 52.73 million reads per library (Table 1). Approximately 89% of the quality filtered reads from each RNA-Seq replicate were aligned to the *Brachypodium* v3.1 genome. A summary of read counts for all libraries and the percentage of reads mapped to the *Brachypodium* genome for the biological replicates of the WT and TR4, TR6 and TR7 lines under control (H_2_O) and long-term salt (SALT) conditions is presented in Table 1. A heatmap of all differentially expressed genes with log_2_ fold changes less than − 2 or greater than 2 is shown in Supplementary Figure 1 (Additional file 2). This heatmap shows distinct branches for the control and salt treatments, and for WT and TR lines under control conditions.

**Table 1:** Information on counts and percentage of reads mapped to the *Brachypodium* reference genome V3.1 in each of the libraries

		Replicate 1		Replicate 2		Replicate 3	
Sample	Treatment	Sequences	Alignment Percent	Sequences	Alignment Percent	Sequences	Alignment Percent
TR4	H2O	53,249,542	88.30%	52,359,476	90.82%	45,775,614	88.02%
TR4	SALT	48,944,606	91.12%	57,529,036	89.53%	38,814,320	90.97%
TR6	H2O	38,114,942	86.69%	40,126,408	89.97%	NA	NA
TR6	SALT	45,966,688	89.27%	48,999,138	85.98%	NA	NA
TR7	H2O	57,330,182	88.78%	56,880,752	87.76%	54,955,330	90.37%
TR7	SALT	57,503,736	87.18%	58,388,176	90.83%	39,674,666	87.78%
WT	H2O	76,734,506	91.64%	80,661,516	91.29%	NA	NA
WT	SALT	47,626,852	91.63%	55,025,324	91.53%	NA	NA

TR4: BdbZIP26:GFP transgenic line 4; TR6: BdbZIP26:GFP transgenic line 6; TR7: BdbZIP26:GFP transgenic line7; WT: wild type *Brachypodium distachyon*. Water is the control treatment and salt is the long-term salt treatment (100 mM NaCl for 3 wks)

### Gene expression analysis of WT and transgenic lines (TR) under control conditions

**Fig. 3:**
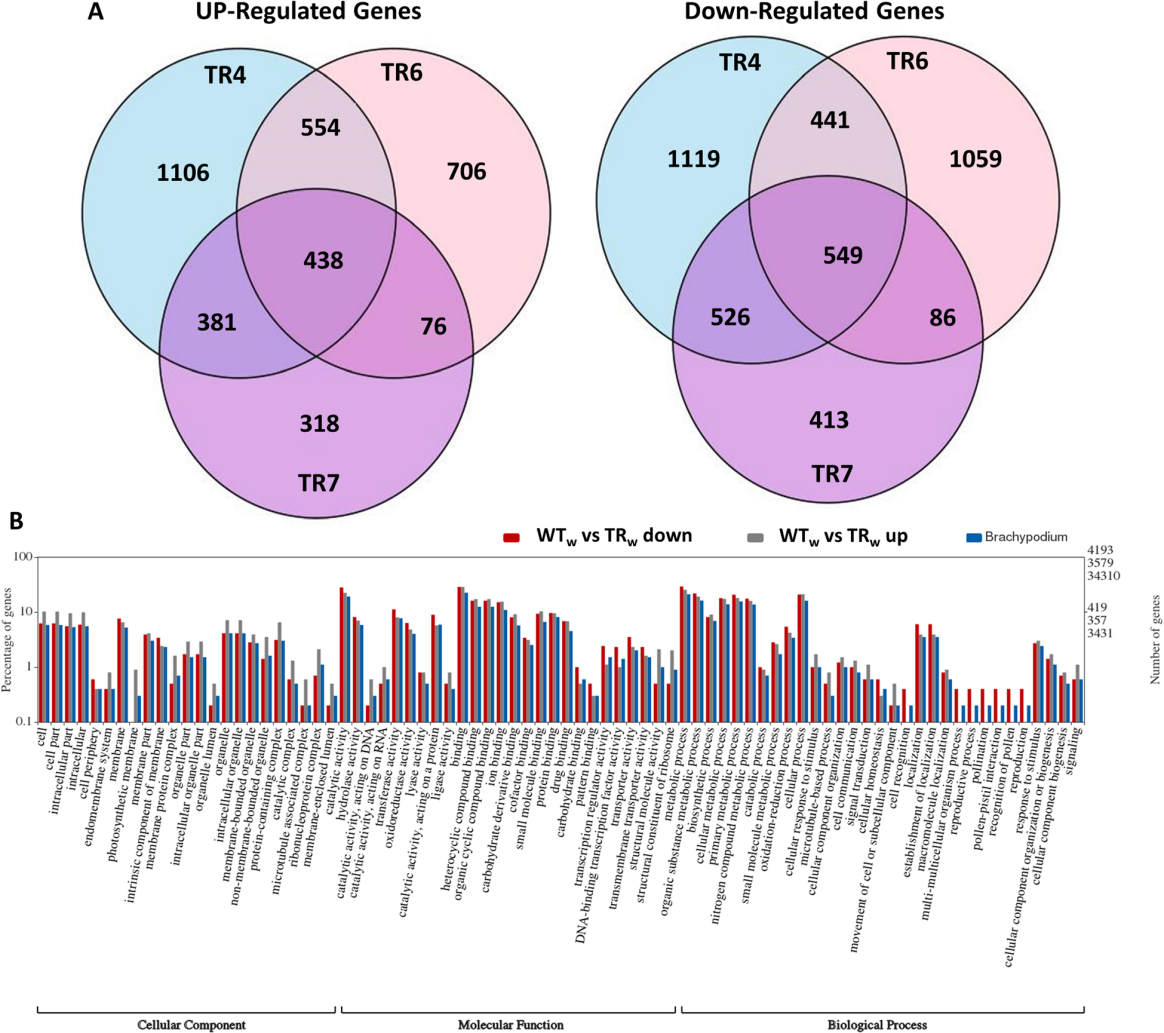
Analysis of gene expression differences between wild type and bZIP26:GFP over-expressing lines under control conditions. **a** Venn diagrams for the up- and down-regulated differentially expressed genes between wild type (WT) and transgenic (TR) lines 4, 6 and 7; **b** Gene Ontology analysis of genes differentially expressed between WT and TR4, TR6, and TR7 under control conditions

A comparison of gene expression between WT and all TR lines was performed. Overall, there were 7772 genes identified that were differentially expressed in the TR lines compared to the WT (4193 down- and 3579 up-regulated) when grown under control conditions (Additional file 3: Table S2). Of the 3579 up-regulated genes identified in the TR and WT comparison, there were 438 genes shared among all three transgenic lines. A three-way Venn diagram of the up- and down-regulated genes in TR compared to WT is displayed in Fig. 3a. Of the 4193 genes differentially down-regulated in the TR lines compared to WT, there were 549 genes shared among the three transgenic lines. Transgenic line 7 had the lowest number of differentially expressed genes overall (2787 genes; with 1213 up- and 1574 down-regulated) when compared to WT under normal conditions (Fig. 3a). Transgenic line 4 had 5114 differentially expressed genes (2479 up- and 2635 down-regulated) and TR6 had 3909 genes differentially expressed (1774 up- and 2135 down-regulated) compared to WT under control conditions.

To gain a better understanding of the categories of genes that were differentially regulated between the TR and WT lines, the WEGO (Web Gene Ontology Annotation Plot) program was used to analyze the GO annotations of the differentially expressed genes in cellular component, molecular function and biological processes categories. WEGO plots of the over- and under-represented GO terms were displayed, along with the number and percent of genes in each of those categories for the *Brachypodium* transcriptome. The categories with significant up- and down-regulated differentially expressed genes (DEGs) between WT plants and TR4, TR6 and TR7 lines, as well as the total number (%) of genes in each category for *Brachypodium* are presented in Fig. 3b. In the cellular component category, most of the sub-categories that were over represented with genes up-regulated in TR compared to WT were associated with cell parts, membranes (endo-, photosynthetic, protein complex), organelles (intracellular, membrane bound, nonmembrane bound), and various complexes (protein, microtubule associated, catalytic and ribonucleoprotein). Interestingly, down-regulated genes were under-represented in the photosynthetic membrane, organelle lumen, membrane protein complex and enclosed lumen membrane subcategories. In molecular function categories, upregulated genes were slightly over-represented in catalytic (hydrolase, DNA, RNA), oxidoreductase, lyase, ligase, binding to various compounds, structural molecule activity and structural constituent of ribosome subcategories, and only slightly under-represented in carbohydrate and DNA-binding transcription factor activity sub-categories. Down-regulated genes in the molecular function category were generally slightly over-represented in most sub-categories, but under-represented in the catalytic activity (acting on RNA or DNA), structural molecule activity and structural constituent of ribosome subcategories. For biological processes, many sub-categories were over-represented slightly for both up- and down-regulated genes including metabolic, biosynthetic, and catabolic processes, response to stimulus, and biogenesis of cellular components subcategories. In the reproduction sub-category, there were no up-regulated genes and the down-regulated genes were over-represented. Signaling, biogenesis, and cellular response to stimulus sub-categories were slightly over-represented for up-regulated genes.

### Genes differentially expressed in transgenic lines compared to WT under control conditions

To further investigate how over-expression of bZIP26 modulates gene expression to effect the changes observed in TR lines compared to WT, we analyzed up- and down-regulated genes present in at least two of the transgenic lines. Based on the GO term categories over/under represented in Fig. 2, we did key word searches to identify potential signaling, biosynthetic and metabolic pathways differentially regulated in the transgenic lines compared to the WT (Table 2). The total number of genes up- and down-regulated in at least two of the transgenic lines compared to WT was 1449 and 1604, respectively. Of those genes, there were 347 up- and 221 down-regulated genes that were without functional annotation (Table 2). Based on the WEGO charts in Fig. 3, one of the GO sub-categories over-represented was the response to stimulus, and this includes genes related to perception, signaling, and responses to different stresses. In biotic and abiotic stress responses, plants often use Ca^+ 2^ as a secondary messenger, and calcium/calmodulin binding proteins/kinases and calcineurin B-like proteins and their interacting kinases as sensor and signaling proteins ([23] and references within, [24]). A query of the up and down DEGs using ‘calcium’, ‘calmodulin’ and ‘calcineurin’ revealed 32 calcium/calmodulin/calcineurin genes that were differentially down-regulated and only six genes that were differentially up-regulated in TR lines when compared to WT (Table 2). Calmodulin binding proteins/kinases were most highly represented in these down-regulated genes, but there were also calcium-dependent protein kinases and EF hand calcium-binding proteins that were down-regulated. Calcium-dependent lipid-binding proteins, which are thought to be involved in signal transduction and vesicle trafficking [25], but may also negatively regulate defense responses [26] and cell death [27], were present in both the down- and up-regulated DEGs.

**Table 2:** Functional analysis of gene classes differentially regulated in at least two of the transgenic lines compared to the WT under control conditions

	UP	DOWN
Total genes differentially expressed	1449	1604
Genes not annotated	347	221
	Differentially Expressed Genes	
Category Search	UP	DOWN
Kinases	75	140
Phosphatases	12	24
Ca/Calmodulin related	6	32
Oxidases/reductases	26	31
Cytochrome P450s + others	9	22
Tetra- Penta- tricopeptide repeats	30	12
Transcription factors/DNA binding	18	45
Myb domain proteins	9	9
Ribosome	41	9
Photosynthesis/Photosystem	31	6
Disease	52	16
NB-ARC domain-containing	34	12
Dirigent	5	0
Methyltransferases	16	12
Ubiquitin and F- and U-box related	30	69
NAC domain containing protein	3	15
Hormone related	14	36
Transporters	18	56

Including the calcium related protein kinases mentioned above, there were 75 up- and 140 down-regulated protein kinases differentially expressed in the transgenic plants compared to the WT (Table 2). Various classes of kinases, such as the leucine-rich repeats, leucine-rich receptor-like protein kinases, mitogen-activated protein kinases (MAPK), and MAPK KKs were present in both the up- and down-regulated DEGs. The lectin related protein kinases, which are involved in hormone signaling and biotic and abiotic stress perception [28], were more highly represented in the down-regulated DEGs. Cysteine-rich receptor-like kinases, which are induced in response to multiple abiotic stresses and involved in pathogen responses and cell death [29, 30], were more highly represented in the down-regulated DEGs. Several other types of protein kinases present only in the down-regulated DEGs included ‘U-box domain-containing protein kinases’ possibly involved in ubiquitination and subsequent degradation of target proteins [31]; ‘wall-associated kinases’, which are induced by hormones and wounding, and are involved in disease resistance signaling [32, 33]; and BAK1 kinases involved in brassinosteroid signaling [34, 35] and pathogen responses [36].

Plant protein phosphatases modulate the action of protein kinases and are an integral part of the phosphoregulatory signaling system [37]. Protein phosphatases 2A and 2C, which are key players in signal transduction [37], were more prevalent in the down-regulated DEGs. Interestingly, Bradi2g54810, a highly ABA-induced PP2C gene 3, was down regulated in the transgenic lines compared to WT, while another gene, “related to ABI3/VP1 2” (RAV1; Bradi2g02710) was up-regulated. RAV1 is considered to be a negative regulator of growth and RAV1 gene expression is repressed by brassinolide. Interestingly, RAV1 over-expression results in insensitivity to ABA [38]. Other hormone related genes in the up-regulated DEGs included 1-amino-cyclopropane-1-carboxylate synthase 7 (increased ~ 36-fold), the rate limiting enzyme for ethylene biosynthesis [39]. Additionally, there were several auxin-responsive genes (‘SAUR-like auxin-responsive protein family’ and ‘Auxin-induced Root cultures 1’), which were upregulated [40, 41]. Other hormone related genes that were down regulated in the TR lines included the gene encoding 9-cis-epoxycarotenoid dioxygenase 9, which is a key enzyme in ABA biosynthesis [42], and a gene encoding the jasmonate-zim-domain protein 11, which is an integral component of the JA signaling pathway [43].

Another class of ‘respond to stimulus’ proteins include proteins produced by plants in response to pathogens. A heatmap of 163 genes classified as pathogen/disease resistance related and differentially expressed with log_2_ fold changes less than − 2 and greater than 2 is shown in Supplementary Figure 2 (Additional file 4). In this heatmap, the WT and TR6 form a cluster separate from TR4 and TR7, which is congruent with TR6 phenotype being less different from the WT than the other two transgenic lines. Included in this heatmap are R proteins, which have a nucleotide binding site and a leucine rich repeat (NBS-LRR) [44]. Disease resistance genes with NB-ARC domains and/or LRR domains and disease resistance-responsive (dirigent-like proteins) [45] were more prevalent in the up-regulated DEGs (Table 2), while an NPR1-like protein (Nonexpressor of Pathogen-Related Genes 1-like), which is also important in plant pathogen interactions [46], was only present in the down-regulated DEGS. There were several genes encoding members of the ‘MATE efflux protein family’ that were significantly down-regulated. Members of this family have been associated with disease resistance in Arabidopsis, and are involved in the synthesis and transport of salicylic acid out of the chloroplast [47–49]. Three thionin genes, which encode antimicrobial peptides [50], were found in the up-regulated genes, with one present in the down-regulated genes.

The penta- and tetra-tricopeptide repeat (PPR, TPR) proteins function mainly in the chloroplast and mitochondria where they can bind to RNA transcripts and influence their expression by stabilizing RNA transcripts, modulating translation, and functioning in transcription and translation of organellar genes [51, 52]. Tetratricopeptide repeats are essential in thylakoid membrane biogenesis [53], and a tetratricopeptide repeat, Pyg7, was shown to be involved in photosystem I (PSI) stability and essential for PSI assembly [54]. Interestingly the penta- and tetra-tricopeptide repeat DEGs were much more prevalent in the differentially up-regulated genes than in the down-regulated genes (Table 2). In addition to the penta- tetra-tricopeptide repeat genes that increase the stability of the photosystem, there were many genes encoding photosystem II reaction center proteins, which were much more highly represented in the up-regulated DEGs.

There are many different mechanisms that result in differential gene expression. One that is probably less commonly thought of is the role ribosomal proteins have in post-transcriptional and translational regulation [55–57]. The differential expression of specific ribosomal proteins in response to abiotic and biotic stress has been previously reported [55, 56, 58, 59]. Interestingly, in this study, ribosomal proteins were more highly represented in the up-regulated (41 up-regulated, nine down-regulated) DEGs. Genes encoding ribosomal proteins and photosynthesis related proteins were also the most enriched in the cellular component category.

Transcription factors are also important for regulating stress responsive gene expression. Of the 56 families of transcription factors, there are several transcription factor families that are more commonly associated with stress responses including MYB, WRKY, bZIP, NAC and AP2-ERF [60]. A heatmap of 213 genes classified as transcription factors and differentially expressed with log_2_ fold changes less than − 2 or greater than 2 is shown in Supplementary Figure 3 (Additional file 5). This heatmap shows clustering of the TR genotypes in a clade separate from the WT under control conditions, and shows a closer relationship between TR4 and TR7, which is consistent with the phenotypes observed. Of the 63 transcription factors differentially expressed between control and at least two of the three transgenic lines, 45 of these were down regulated, possibly suggesting that the plants were selectively reducing metabolism to survive. Represented in these down regulated genes were four in each of the bZIP, Heat Shock, and GRAS transcription factor families. In the WRKY and MYB transcription factor families, 15 and 8 members were down regulated, respectively. The WRKY and MYB families of transcription factors are important regulators in many plant processes, including plant development, hormone signaling and responses to multiple biotic and abiotic stresses [9, 61, 62]. The MYB transcription factors also interact with BHLH (basic helix-loop-helix) proteins [63], and there were eight members of this family, which were also differentially down-regulated. Four bZIP transcription factors were present in each of the up- and down-regulated DEG categories, which is interesting because bZIP transcription factors are known to homo- and/or heterodimerize with other bZIP proteins. GRAS proteins are important in hormone (gibberellin and jasmonate) signaling, phytochrome signaling and meristem development [64, 65], and were present in both the up- (2) and down-regulated (4) DEGs. NAC domain containing proteins, which are involved in various stress signaling pathways and possibly play a role in the interactions between hormones [66, 67], were more highly represented in the down-regulated DEGs (15 down DEGs). The 21 up-regulated transcription factors included members of the MYB (9), bZIP (4), GRAS (2), NAC (3) and WRKY (3) gene families. It is possible that the increased level of differentially down-regulated genes is a result of the plant reallocating its resources to programs that are essential for plant survival.

Interestingly, there were several genes associated with cell cycle and cell death processes that were differentially expressed between the WT and TR lines. One of the up-regulated genes encoded for cyclin D3, and members of this family of cyclins are involved in the G1-to-S transition during cell division and are thought to help regulate cell cycle progression in response to nutrient and hormone signals [68]. Other up-regulated genes encoded small GTPases, which are important in cell cycle progression, and interacting genes such as, ‘Ran BP2/NZF zinc finger-like superfamily protein’ [69] and ‘RHO-related protein from plants 9’ [70]. Interestingly, genes encoding proteins responsible for transitioning between inactive to active forms of GTPases such as a RHO guanine exchange factor 3 and a Rab GTPase activator family protein were down-regulated [69, 70]. Several other genes associated with cell death were present in the down-regulated DEGs. These genes encoded a Bax inhibitor-1 protein, which inhibits the cell death provoking BAX protein; and a member of the Bcl-2 associated anthanogene (BAG) protein family, which are thought to play a key role in promoting cell survival (reviewed in [71]). The ability to regulate the cell cycle and cell death is probably essential for the transgenic lines to allocate resources in order to survive.

### Comparison of transgenic lines to WT plants under salt stress

**Fig. 4:**
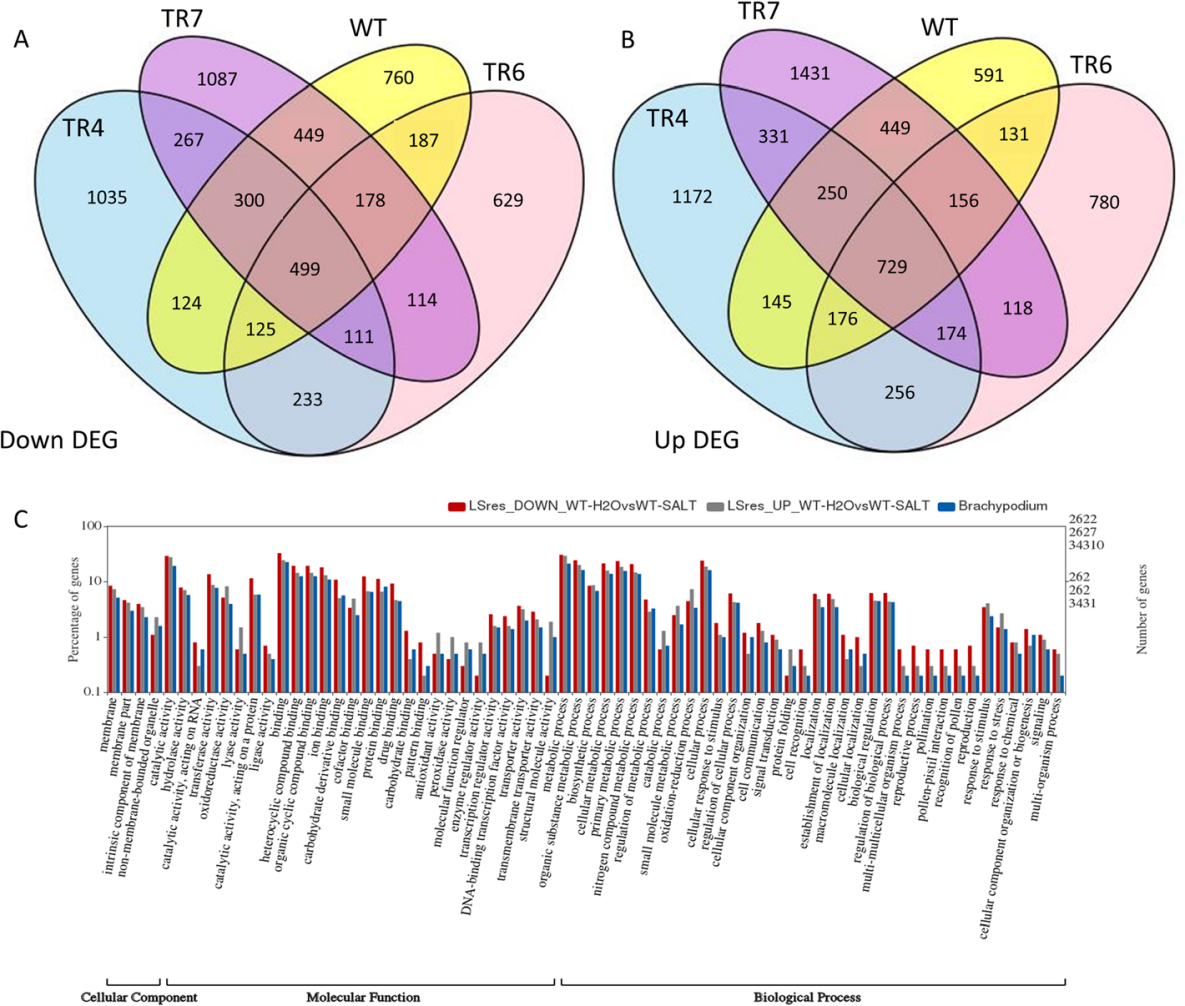
Analysis of gene expression in wild type and bZIP26:GFP transgenic lines (TR) when exposed to salinity stress. Venn diagrams (**a** and **b**) for the up- and down-regulated differentially expressed genes in wild type (WT) and TR4, TR6 and TR7; **c** Gene Ontology analysis for up- and down-regulated genes in WT in response to salt stress

To gain a deeper understanding of the differences between the transgenic lines and WT, we looked at the effect of long-term salinity stress on plant growth. After 3 weeks of salt exposure (100 mM NaCl), both the WT and the transgenic lines were stunted and were starting to show browning of leaf tips, which seemed more pronounced in the WT plants (Fig. 2e). Data for the genes included in the Venns for up and down regulated genes in response to long term salt stress are listed in Table S3 (Additional file 6). A Venn diagram of shared and unique up and down regulated genes in TR4, TR6, TR7 and WT in response to salt stress is shown in Fig. 4a (down) and b (Up). A summary of the DEGs in response to salt stress is presented in Table 3. Of the 12,987 genes differentially regulated in response to long term salt stress, 53% of the genes were up-regulated and 47% were down-regulated in treated plants compared to plants grown under control conditions. Approximately 11% of the up- and 8% of the down-regulated genes were shared between all transgenic lines and the WT (Table 3). Only 2.5% of the up- and 1.8% of the down-regulated DEGs were shared among all three of the transgenic lines and absent from the WT. Approximately an additional 10% of the up- and down-regulated genes were present only in two of the three transgenic lines and not in the WT (Table 3). If one looks at the proportion of unique shared DEGs between each transgenic line and the WT, between 39 and 47% of the DEGs in the individual transgenic lines are shared with the WT, while 38–60% of the WT DEGs are shared with each TR line DEGs. While TR7 shares a greater proportion of the WT DEGs, this may be due to the larger overall number of DEGs associated with this transgenic line. TR6 appears to be most similar to WT, as 47% of the total up- and down-regulated TR6 DEGs were also present in the WT’s DEGs. TR4 is probably most different from the WT, with roughly 40% of both up- and down-regulated DEGs being shared with the WT DEGs. TR4 was also the most dwarfed of the transgenic lines.

**Table 3:**
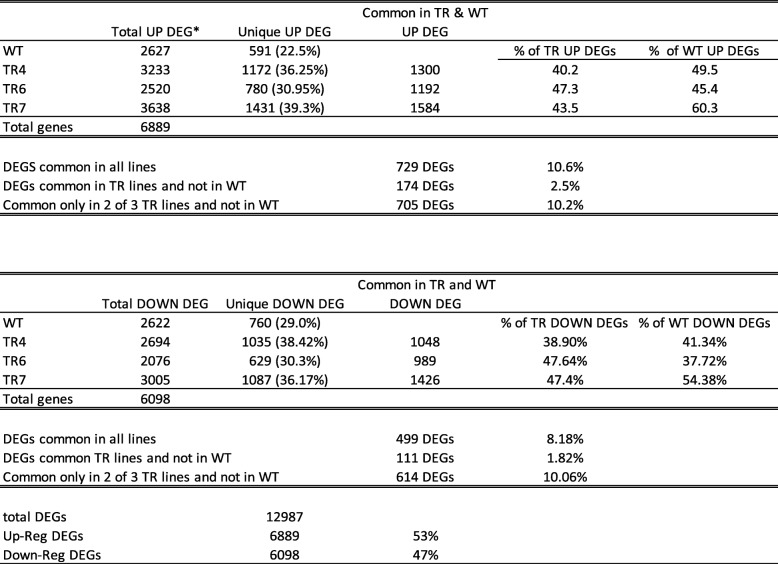
Summary of DEG in WT and TG lines exposed to salt stress

*DEG Differentially Expressed Genes, *UP* Up-regulated, *DOWN* Down-regulated, *TR* Transgenic line, *WT* Wild type

To gain a better understanding of the types of genes that were differentially regulated in the WT and TR plants in response to long-term salt, WEGO program was used to analyze the Gene Ontology (GO) annotations of the differentially expressed genes in cellular component, molecular function and biological process categories. The sub-categories with significant up- and down-regulated DEGs between control and long-term salt treated WT plants, along with the total number (%) of genes in each sub-category in the *Brachypodium* transcriptome are presented in Fig. 4c. In WT plants, both up- and down-regulated DEGs were over-represented in membrane parts sub-category; while up- and down-regulated genes in the non-membrane bound organelle sub-category were over- and under-represented, respectively. Several groups in the molecular function category were over-represented in both down- and up-regulated DEGs, such as binding, transporter, catalytic activity, and ligase; while up-regulated genes were over-represented in lyase, antioxidant, peroxidase, and structural molecule categories; and down-regulated genes were over-represented in catalytic (RNA and protein), ligase, binding (carbohydrate, small molecule, protein) and transcription. In terms of biological process, many sub-categories were slightly over-represented in both up- and down-regulated DEGs. Sub-categories with an over-representation of down-regulated genes included cellular response to stimulus, cellular processes/component, cell recognition and localization and reproduction. The up-regulated DEGs were over-represented in catabolic process, protein folding, response to stress and oxidation-reduction categories, all processes related to abiotic stress responses. Many of the same trends were seen in the transgenic lines (data not shown), but with a greater representation of the cellular and organelle component sub-categories with increased up- and down-regulated DEGs in the transgenic plants. The up-regulated DEGs related to structural molecule activity were over-represented in the WT, but under-represented in the TR lines. While the up-regulated DEGs in the peroxidase category were highly over-represented in the WT plants, they were not enriched in the transgenic lines. Many of the categories over- and under-represented are what one would expect in plants responding to abiotic stress.

Since TR plants were stunted under control conditions and similar to the WT plants under salt stress, we thought it would be interesting to see if genes differentially expressed between the WT and TR lines under control conditions would overlap with genes uniquely differentially expressed in the WT under salt stress. Of the 438 genes that were up-regulated in all three transgenic lines compared to WT under control conditions, 202 of these genes were also up-regulated in the WT under long term salt conditions. Similarly, of the 549 genes that were down-regulated in all three transgenic lines compare to WT under control conditions, 226 were also down-regulated in WT plants exposed to long term salt stress, suggesting that these overlapping genes play a role in the salt stress response. This also means that there are 323 down- and 236 up-regulated DEGs in TR lines compared to the WT that were not responsive to long-term salt stress. The genes present in all three TR lines compared to the WT, that were not present in the WT long-term salt stress DEGs, were examined further using the Panther Classification System [72] to identify the types of genes present. The down-regulated DEGs were related to catalytic activity, binding and transporter activity, with the main protein classes being hydrolase, oxidoreductase, transferase, and transporter. The up-regulated DEGs were also categorized mainly into the catalytic and binding activity categories, with protein classes related to nucleic acid binding, hydrolase, transferase, transporter, and enzyme modulator. There were no major shifts in gene classification between the genes in common with the salt treated WT plants and the total up- and down-regulated DEGs.

### Quantitative RT-PCR validation of RNA-sequencing data

To validate the RNA-seq results, qRT-PCR was used to analyze DEGs identified in the RNA-Seq results. Genes that were differentially expressed between WT and TR lines under control or in response to salt stress were chosen for analysis. The comparison between the RNA-Seq and qRT-PCR results are shown in Figure S4 (Additional file 7). There was a general agreement between the RNA-seq and qRT-PCR results for all genes analyzed validating the RNA-seq library.

## Discussion

Over the last 20 years, there has been an increased effort to utilize biotechnology for improving stress tolerance in crop species. Over-expression of bZIP transcription factors has been reported to increase tolerance to drought, salinity, cold, and/or pathogens [3]. The bZIP27 (same as our BZIP26) transcription factor was previously found to be responsive to cold, heat, polyethylene glycol, sodium chloride, salicylic acid, benzylaminopurine and abscisic acid [12], suggesting that it may play a key role in stress responses. In this paper, we investigate the possibility of over-expressing bZIP26 for improved stress tolerance, examine the effects of constitutively over-expressing the bZIP26 transcription factor on the plant phenotype, and compare transcriptomes in WT and bZIP26 transgenic lines under control and salinity stress conditions. A BLAST search at NCBI to identify the closest Arabidopsis homologue to the Bradi1g76690 protein indicated that it shared 46% homology to Arabidopsis bZIP29 (AAK84220.1). The Arabidopsis bZIP29 protein was identified during an interactomics study of core cell cycle machinery in Arabidopsis [73], and was later shown to be expressed in proliferating tissues and to be involved in cell wall organization [74]. In our study, the stunted growth observed in tissue culture was relieved when GA was added to the medium and TR lines grew better than WT in the presence of ABA. This suggests that hormones are involved in the reduced stature observed in the transgenic plants. This stunted phenotype is consistent with what Van Leen et al. [74] observed in Arabidopsis, as when they overexpressed the bZIP29 gene in Arabidopsis, the plants had impaired leaf growth and fewer leaves.

**Fig. 5:**
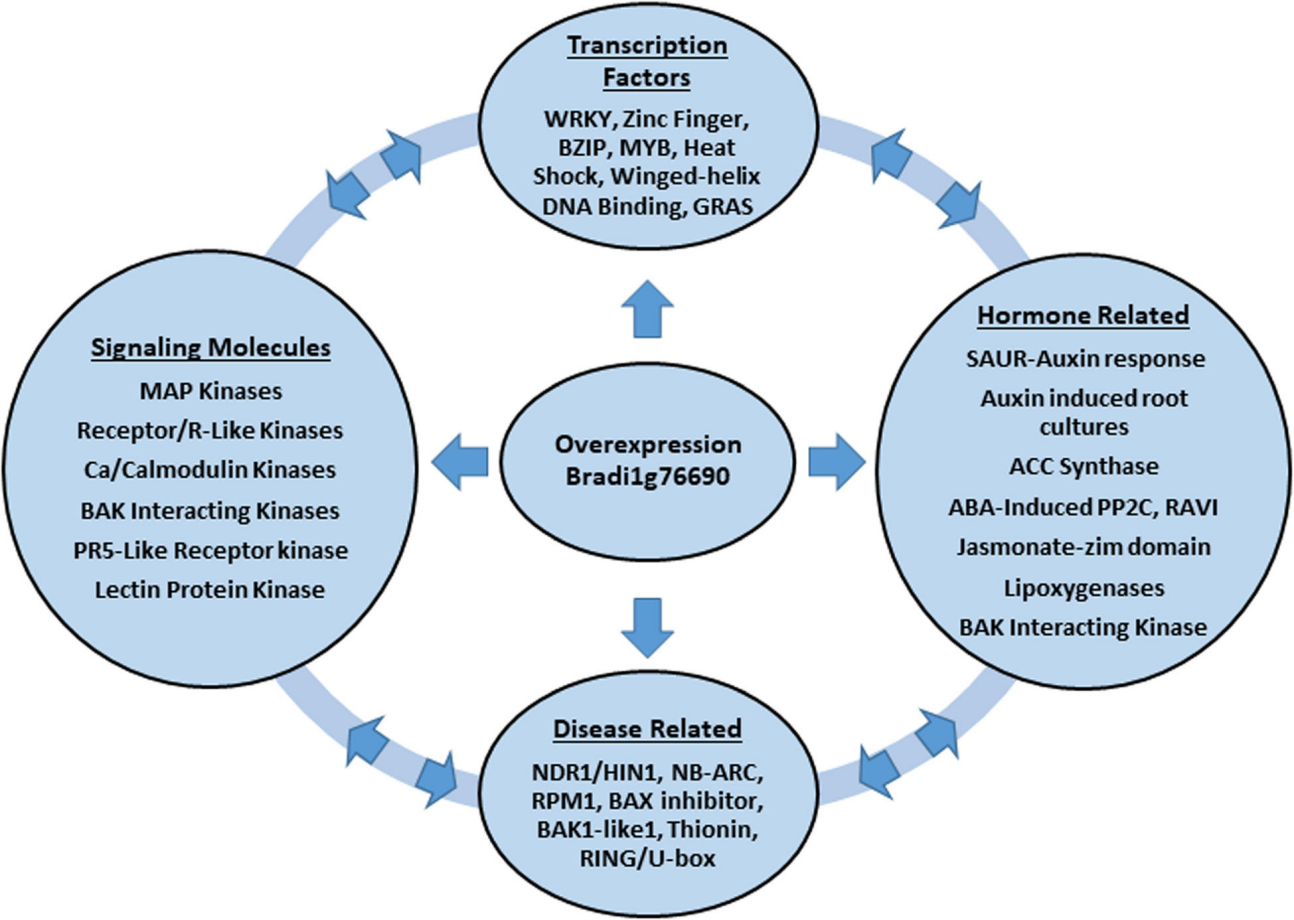
Summary of genes affected by overexpressing bZIP26:GFP in *Brachypodium*. Genes depicted in each category were differentially expressed (log_2_ fold changes > 2 or < − 2) in at least two of the three transgenic lines. Abbreviations: Ca, Calcium; R, receptor; BAK, BRI1-associated kinase 1; PR5, Pathogenesis Related; ACC, 1-amino-cyclopropane-1-carboxylate synthase; ABA, Abscisic Acid; SAUR, Small auxin-up RNA; PP2C, Protein phosphatase 2C; RAV, Related to ABI3/VP1

To further examine the stunted phenotype in the transgenic plants, we compared the transcriptome of WT plants and TR lines under control conditions. Transcriptome analysis revealed that there was a broad range of genes affected by the overexpression of bZIP26. A summary of genes that were differentially expressed between WT and transgenic lines is presented in Fig. 5. In our analysis, we found a number of DEGs involved in hormone biosynthesis, perception and response. Interestingly, RNA-Seq revealed the down regulation of several genes encoding ABI-1-like proteins. ABI proteins were first discovered when screening for ABA insensitive mutants that could grow in the presence of ABA [75]. We also found that a gene in the ABA biosynthetic pathway, nine-cis-epoxycarotenoid dioxygenase, was down-regulated in transgenic plants. This is congruent with the ability of the transgenic seedlings to grow on ABA. Interestingly, one of the genes encoding ACC synthase, which is important for ethylene biosynthesis, showed a significant up-regulation in the transgenic lines. Ethylene is known to be induced in response to biotic and abiotic stresses [76, 77]. Surprisingly, while the gene for ethylene production was up-regulated, some ethylene response elements were down regulated. It is possible that these differences in expression could be due to tissue specificity (root crown/roots vs shoots) of expression, and it would probably be more informative to investigate the tissue specific expression of these genes. Ethylene production may also cause a redistribution of auxin within the plant [78]. In this study, auxin responsive genes and several auxin efflux carriers were present in the up- and the down-regulated genes suggesting a possible change in auxin distribution. Several jasmonate-zim-domain protein genes were down regulated in the transgenic lines. These proteins are part of the jasmonate signaling pathway, but are also thought to be involved in defense responses, with mutants showing increased sensitivity to various pathogens and also showing reduced vegetative growth [79]. It is evident that the ethylene, auxin, ABA, and JA biosynthesis and/or signaling pathways were altered in the transgenic plants.

A bZIP26 protein BLAST search of *Oryza sativa* (organism) revealed the closest protein, which shared 58.8% homology to bZIP26, was the transcription factor RF2b-like protein (XP_015627932.1). The RF2a and RF2b genes in rice were previously shown to regulate the expression of the rice tungro bacilliform virus [20, 21]. Since bZIP proteins operate as dimers, it was interesting to find that one of the up-regulated bZIP genes (Bradi4g22130) in the transgenic lines was closely related to an RF2-like gene in wheat that is induced by stripe rust. There were many disease resistance genes that were up-regulated in the transgenic lines, suggesting that bZIP26 could be involved in biotic stress responses. Interestingly, genes encoding WRKY transcription factors and a gene encoding a TGAL3-type bZIP transcription factor, which may be involved in the regulation of the NPR (nonexpressor of pathogenesis-related) genes [80], were negatively regulated in the transgenic plants; as were suppressor of npr-1 constitutive 4 (SNC1) and NPR1-like protein 3 [81]. Many NB-ARC domain-containing disease resistance proteins, dirigent-like proteins and plant thionin genes were up-regulated in the transgenic lines. Plant defense genes are also regulated by ethylene, and this is another possibility for increased expression of disease resistance related genes, since the gene encoding the rate-limiting step in ACC biosynthesis was also up-regulated in transgenic lines. It is possible that these genes are indirectly regulated by bZIP26 or it could be that the stressed plant is inducing these defense genes to protect itself from pathogen attack.

The chloroplast is an important organelle, not only for photosynthesis, but also for amino acid, lipid, fatty acid, vitamin, and plant hormone biosynthesis [82]. It was interesting to see a large number of photosystem II reaction center proteins up regulated in the transgenic line, which suggests plants are working to keep the photosynthetic machinery intact. There were also many ribosomal proteins that were up-regulated in the transgenic lines. The differential expression of ribosomal proteins has been shown previously in water-use-efficient plants under drought conditions [83]. There were also many down-regulated DEGs coding for different cytochrome P450s, which are important for mediating the biosynthesis of defense compounds and for detoxification of toxins [84] and, which have previously been shown to be induced in response to multiple abiotic stresses [85].

The WRKY and NAC domain containing protein transcription factors, which are involved in both abiotic and biotic stress responses, were mostly down regulated in the transgenic plants. Many genes encoding proteins involved in stress signaling were present in both the up- and down-regulated DEGS, but were present in greater numbers in the down-regulated genes, for example, kinases, phosphatases, calcium/calmodulin related genes, transcription factors, and transporters. It seems evident that many stress related pathways are being activated in the TR lines, but it is difficult to discern the underlying mechanisms effecting these changes and how they may affect the stature of the plant.

## Conclusion

Over the last 20 years, there has been an increased effort to utilize biotechnology for improving stress tolerance in crop species. In this paper we investigated the effects of overexpressing the bZIP26 transcription factor in *Brachypodium* and found that it affected growth in a negative way, producing plants with a reduced stature and activating multiple stress related pathways. The transgenic plants showed altered expression of genes involved in pathogen responses and genes in ABA-, auxin-, ethylene-, and JA-related pathways; both of which could contribute to the reduced stature of the plant. In future studies, it would be interesting to examine how these plants respond to pathogens to determine if these pathogen responsive genes would help to defend against certain types of pathogens. Additionally it would be interesting to see if expression of this gene under a stress-inducible promoter could potentially alleviate the negative phenotype observed in this study and increase performance under salinity stress.

### Limitations

While looking at multiple transgenic lines is important due to potential differences in transcriptional profiles as a result of their positional insertion into the genome, this complicates the analysis of the effects of the transgene.

## Methods

### Construction of *Agrobacterium* vector

BdbZIP26 nomenclature (protein name, Gene ID [Bradi1g76690], and nucleotide sequence) was obtained from the transcription factor database found at http://www.grassius.org. Primers for amplifying the BdbZIP26 gene for In-Fusion cloning into a modified pART vector with expression under the control of a ZmUbi1 promoter [86] containing a C-terminal GFP tag [87–89] were designed using the Clontech’s web based In-Fusion cloning primer design tool. Forward (5′- TCGACTCTAGAGGATCCATGGACTCGCGCGCGG) and reverse (5′- TGCTCACCATGGATCCCTCTCTGGGCTCATGGTTG) primers were used to amplify the coding region of bZIP26 from cDNA derived from *B. distachyon* plants. Vector preparation and In-Fusion cloning reactions were performed following the manufacturer’s recommendations, and the product was transformed into competent DH5α cells. The plasmid was purified using Qiagen midiprep columns, sequenced to verify the construct, and then transformed into *Agrobacterium tumefaciens* AGL1 [90]. A diagram of the vector is shown in Fig. 1A.

### *Brachypodium* transformation, growing conditions and identification of homozygous lines

*Brachypodium* Bd21–3 seeds were obtained from Dr. John Vogel (USDA ARS, Albany CA). *Brachypodium* transformation with *Agrobacterium* AGL1 containing Ubi:bZIP26:GFP was performed as previously described [91, 92]. Briefly, immature embryos were aseptically dissected from seeds, placed on Callus Induction Media (CIM), and cultures were placed in the dark. The yellow compact embryogenic callus was subcultured onto the same medium every 2–3 weeks to enrich for embryogenic callus. After about six-eight weeks, the embryogenic callus was suspended in liquid LS medium [93] with 200 μM acetosyringone (Sigma; MO), 2.5 mg/L dichlorophenoxyacetic acid (Sigma; MO), 0.1% symperonic PE/F68 (Sigma; MO), and AGL1 with the pART bZIP26:GFP vector (O.D. of ~ 0.6) for about 15 min. The liquid was then removed, the embryogenic calli were moved to dry sterile Whatman filter paper, and placed in the dark for 3 days. Following co-cultivation, the embryogenic callus was transferred to CIM media containing 150 mg/L timentin to prevent *Agrobacterium* growth. One week later, the callus was transferred to CIM media with 150 mg/L timentin and 40 mg/L hygromycin to select for transformed calli. After an additional 3–5 weeks, transformed (live) callus was moved to regeneration media with 40 mg/L hygromycin, and placed under 16-h light/8-h dark cycles at 28 °C. Regenerating shoots were removed as they developed and placed on MS sucrose medium in magenta boxes under the same conditions. Plantlets were transferred to soil, acclimated under a plastic dome for a week and then grown as described in an earlier paper [16]. Sunshine Growing Mix (Sun Gro Horticulture Inc., Bellevue, WA) was used for all greenhouse plantings. Plants were fertilized with Osmocote Plus 15–9-12 slow release fertilizer. Plants were grown and treated under greenhouse conditions (set at 16 h light and temperature of 24 °C during day and 18 °C night).

To identify transformants, PCR was used to test seedlings for the presence of the hygromycin phosphotransferase gene. Genomic DNA was purified from seedlings using the Rapid One-Step Extraction (ROSE) method [94]. The BioRad Real Time PCR Detection System (Bio-Rad Laboratories; Hercules, CA) was used for PCR analysis with the following program: 95 °C for 3:00 min, followed by 50 cycles of 95 °C for 15 s, 60 °C for 1:00 min following the manufacturer’s recommendations with detection of the FAM fluorophore during the extension cycle. Primers and probes used for q-PCR are: Hyg primer F1020 (GCTTGGTTGACGGCAATTT), Hyg primer R1155 (CCACTATCGGCGAGTACTTCTA) and the probe sequence (5′−/56-FAM/ACACAAATC /ZEN/GCCCGCAGAAGC/3IABkFQ/− 3′). Plants that amplified the hygromycin phosphotransferase gene were grown and seed was collected. Progeny in subsequent generations were tested to identify homozygous lines for further studies. Three transgenic lines (TR4, TR6 and TR7) from independent transformation events with elevated expression of the bZIP26 gene were chosen for further analysis.

### Stress assays

For sequencing experiments, plants were grown in a walk-in growth chamber Conviron model PGW40 with settings of 10 h light and day/night temperatures of 24 °C/ 18 °C. Thirty-one-day-old potted *Brachypodium* plants, with five plants per 500-ml pot, were treated by watering with water (control plants) or with 100 mM sodium chloride for 3 weeks (100 mls NaCl solution/pot the first 3 days, and then 100 mls every other day) to evaluate growth of WT and TR4, TR6 and TR7 under control and salinity stressed conditions. After 3 weeks, samples (comprised of the aerial portions of the plant, root crown and ~ 1 cm of the roots) were collected from control and treated plants, frozen immediately in liquid nitrogen, and stored at − 80 °C until processing.

### Effect of plant hormones on early plant growth

Seeds of WT and transgenic plants were placed on ½ MS (Murashige and Skoog) medium containing 15 g sucrose/L and 1% agar until roots were 2.5–5 mm in length and then transferred to same media with no hormones (control), 20 μM GA3 (Sigma G1025; St. Louis, MO), or 2 μM ABA (Sigma A1049; St. Louis, MO). Seeds were also placed on medium with 2 μM ABA directly, but neither WT nor transgenic plants germinated in the presence of ABA. Plates were placed upright in a Tissue Culture Conviron TC80 Chamber (Conviron; Winnepeg, Canada). Pictures were taken 21 days post-transfer to hormone plates for ABA, 12 days post-transfer for control plants, and 7 days post-transfer for GA treatment. Pictures were taken of representative plates, arranged with three seeds of each genotype, always in order of WT, TR4, TR6, and TR7.

### RNA extraction and quantification

Tissues were ground in liquid nitrogen with a mortar and pestle prior to RNA extraction with the Direct-zol™ RNA MiniPrep kit (Zymo Research; Irvine, CA). Frozen ground tissue was added to TRI reagent, homogenized with the Ultra-Turrax T25 homogenizer (IKA; Wilmington, NC) for 30 s at a setting of 4, and quick frozen at − 80 °C. Samples were thawed, mixed, centrifuged for 2 min at 13000 x g to remove debris, and processed using the manufacturer’s recommended conditions, including the on-column DNase treatment (Zymo Research; Irvine, CA). RNA was quantified on a DS-11 Spectrophotometer (DeNovix; Wilmington, DE), and approximately 3 μg of RNA was used to synthesize first strand cDNA using the Superscript III First Strand Synthesis SuperMix kit (Invitrogen; Carlsbad, CA) following the manufacturer’s recommendations.

### Quantitative reverse transcriptase-PCR (qRT-PCR)

The BioRad iTaq™ Universal SYBR Green Supermix and the BioRad Real Time PCR detection system (Bio-Rad Laboratories; Hercules, CA) were used for qRT-PCR analysis. Primers were designed with QuantiPrime [95] using the website’s default settings. Primer pairs were assessed for amplification efficiency and linearity using a standard curve of amplification products derived from sequential dilutions of a single cDNA. Only primers with good melt curves and within 90–110% efficiency were utilized for quantification studies. The *BdUBC18* gene was used as the reference gene for normalization [96]. Primers used for qRT-PCR and qPCR are listed in Table S1 (Additional file 1). The ΔΔC_T_ value was used for expression analysis as described by Applied Biosystems in their “Guide to Performing Relative Quantitation of Gene Expression Using Real-Time Quantitative PCR” (https://assets.thermofisher.com/TFS-Assets/LSG/manuals/cms_042380.pdf) [97].

### Illumina RNA-sequencing

RNA samples were sequenced at the Oregon State University Center for Genome Research and Biocomputing (OSU CGRB). Three replicates for each sample were prepared using the Wafergen RNA kit, checked for quality on the Agilent Bioanalyzer (Agilent Technologies, CA), and 100 bp paired-end sequences were completed on the Illumina HiSeq 3000 (San Diego, CA).

### Analysis

Sequences were adapter and quality trimmed with Cutadapt with parameters -q 15,10 [98]. Each RNAseq replicate was aligned to the *Brachypodium* v3.1 genome (International *Brachypodium* Initiative, 2010) [99] with HISAT2 [100]. BAM files were manipulated with SAMtools [101]. StringTie [102] was used to quantitate aligned sequences. Differential expression was conducted with DESeq2 [103]. Custom Perl scripts were created to further analyze the results. Over represented GO terms were displayed using WEGO 2.0 [104] using up and down regulated genes along with the *Brachypodium* gene sets. Venn diagrams were constructed using R (R Core Team, 2017) package VennDiagram [105]. Data was transformed in DESeq2 using variance stabilizing transformation (vst). Pheatmap [106] was used to create heatmaps and were clustered using Euclidean distance.

## Supplementary information

**Additional file 1: Table S1.** List of primers for select genes for qRT-PCR expression analysis, and for primer/probes for testing transgenic plants for the hygromicin phosphotransferase gene.

**Additional file 2: Figure S1.** Heatmap of the 3536 genes that pass the differential expression filter of *p*-value 0.05, FDR 0.5, and log_2_ fold changes less than − 2 or greater than 2. Values were transformed with variance stabilizing transformation. Treatments of H_2_O and salt, and genotypes WT or transgenics 4HA, 6FA, 7DA are shown as colored bars. Samples (columns) and genes (rows) are clustered using Euclidean distance and dendograms are shown.

**Additional file 3: Table S2.** Data for Venn of differentially expressed genes in the three transgenic lines compared to the wild type.

**Additional file 4: Figure S2.** Heatmap of the 163 genes that are classified as disease resistance related genes that also pass the differential expression filter of p-value 0.05, FDR 0.5, and log_2_ fold changes less than − 2 or greater than 2. Values were transformed with variance stabilizing transformation. Treatments of H_2_O and salt, and genotypes of WT or transgenics 4HA, 6FA, 7DA are shown as colored bars above the columns. Classifications of disease related gene types are shown under the ‘Classification’ legend heading and are shown to the left of the rows. Samples (columns) are clustered using Euclidean distance and a dendogram is shown.

**Additional file 5: Figure S3.** Heatmap of the 213 genes that are classified as transcription factors that also pass the differential expression filter of p-value 0.05, FDR 0.5, and log_2_ fold changes less than − 2 or greater than 2. Values were transformed with variance stabilizing transformation. Treatments of H_2_O and salt, and genotypes of WT or transgenics 4HA, 6FA, 7DA are shown as colored bars above the columns. Gene classifications of transcription factor types are shown under the ‘Classification’ legend heading and are shown to the left of the rows. Samples (columns) are clustered using Euclidean distance and a dendogram is shown.

**Additional file 6: Table S3.** Data for Venn of genes up regulated in TR4 (1), TR6 (2), TR7 (3) and wild type (WT) when exposed to 100 mM NaCl for 3 weeks.

**Additional file 7: Figure S4.** Quantitative RT-PCR validation of RNA-Seq data. Log_2_ fold change values obtained by qRT-PCR are plotted against the RNA-Seq data. Primers used for qRT-PCR are listed in Supplementary Table 1.

**Additional file 8: Table S4.** Tables of differentially expressed down-regulated genes between wildtype and each transgenic line, and between wildtype control and long term salt treatments.

**Additional file 9: Table S5.** Tables of differentially expressed up-regulated genes between wildtype and each transgenic line, and between wildtype control and long term salt treatments.

## Data Availability

Data: All data discussed in the paper are available in Supplementary Tables, as referenced in the paper. Complete differential expression data for all genotypes are available in Additional files 8 and 9. The seeds for the original plant material used in this research are publically available and can be obtained from the United States Department of Agriculture - Agricultural Research Service National Plant Germplasm System or by contacting the corresponding author.

## Abbreviations

ABA: Abscisic acid
bZIP: Basic leucine zipper
DEG: Differentially expressed genes
GA: Gibberellic acid
GFP: Green fluorescent protein
GO: Gene ontology
qRT-PCR: Quantitative reverse transcription-polymerase chain reaction
NaCl: Sodium chloride
ROS: Reactive oxygen species
TR: Transgenic
WT: Wild type
