# Supplementary figures and images for: Transcriptome analysis of responses in Brachypodium distachyon overexpressing the BdbZIP26 transcription factor
Source: BMC Plant Biol. 2020 Apr 20;20:174. doi: 10.1186/s12870-020-02341-3 (PMC7171782; doi:10.1186/s12870-020-02341-3)

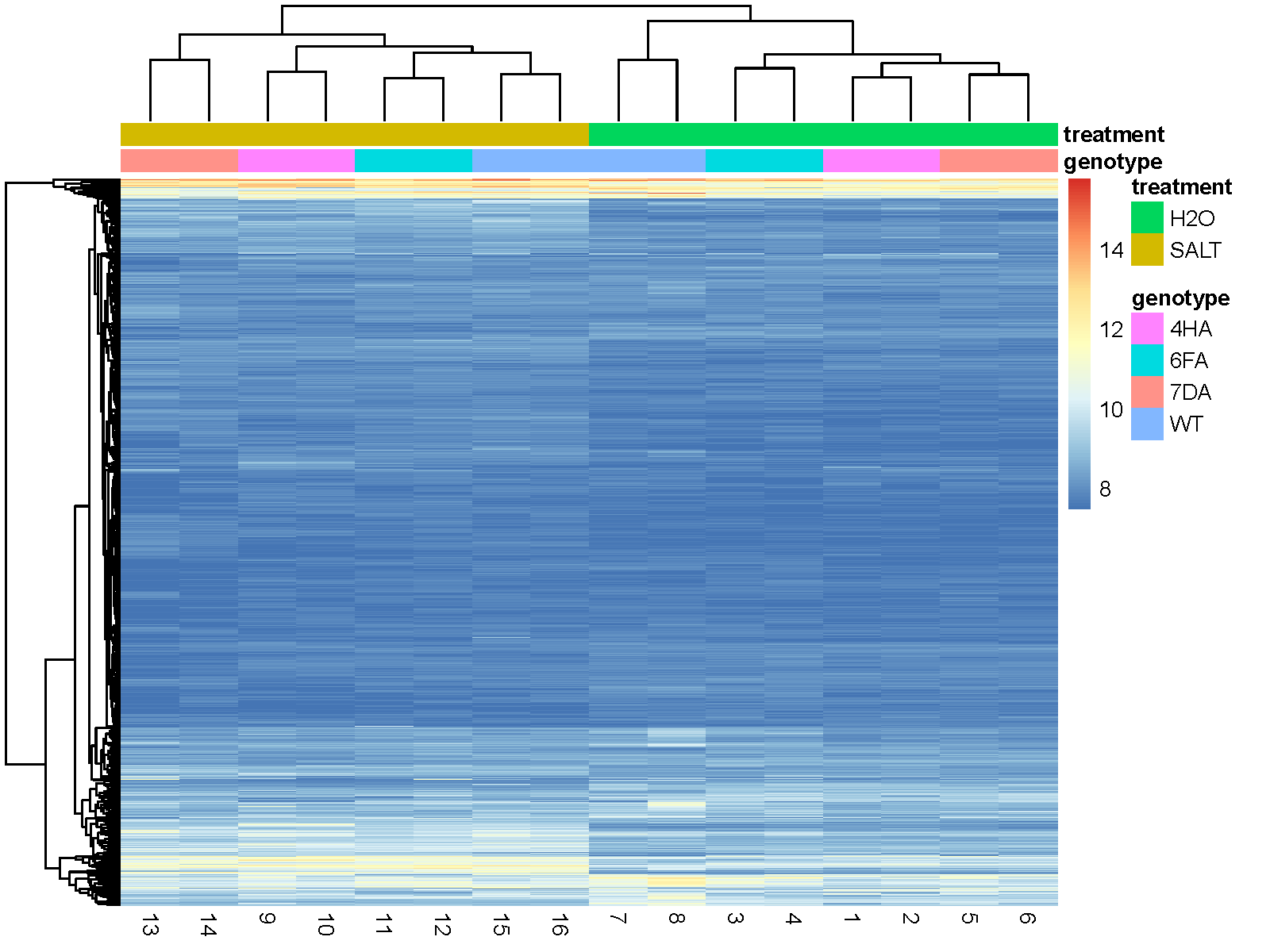

Supplement: Supplementary file 2 — Additional file 2: Figure S1. Heatmap of the 3536 genes that pass the differential expression filter of p-value 0.05, FDR 0.5, and log2 fold changes less than − 2 or greater than 2. Values were transformed with variance stabilizing transformation. Treatments of H2O and salt, and genotypes WT or transgenics 4HA, 6FA, 7DA are shown as colored bars. Samples (columns) and genes (rows) are clustered using Euclidean distance and dendograms are shown. [file 12870_2020_2341_MOESM2_ESM.png]

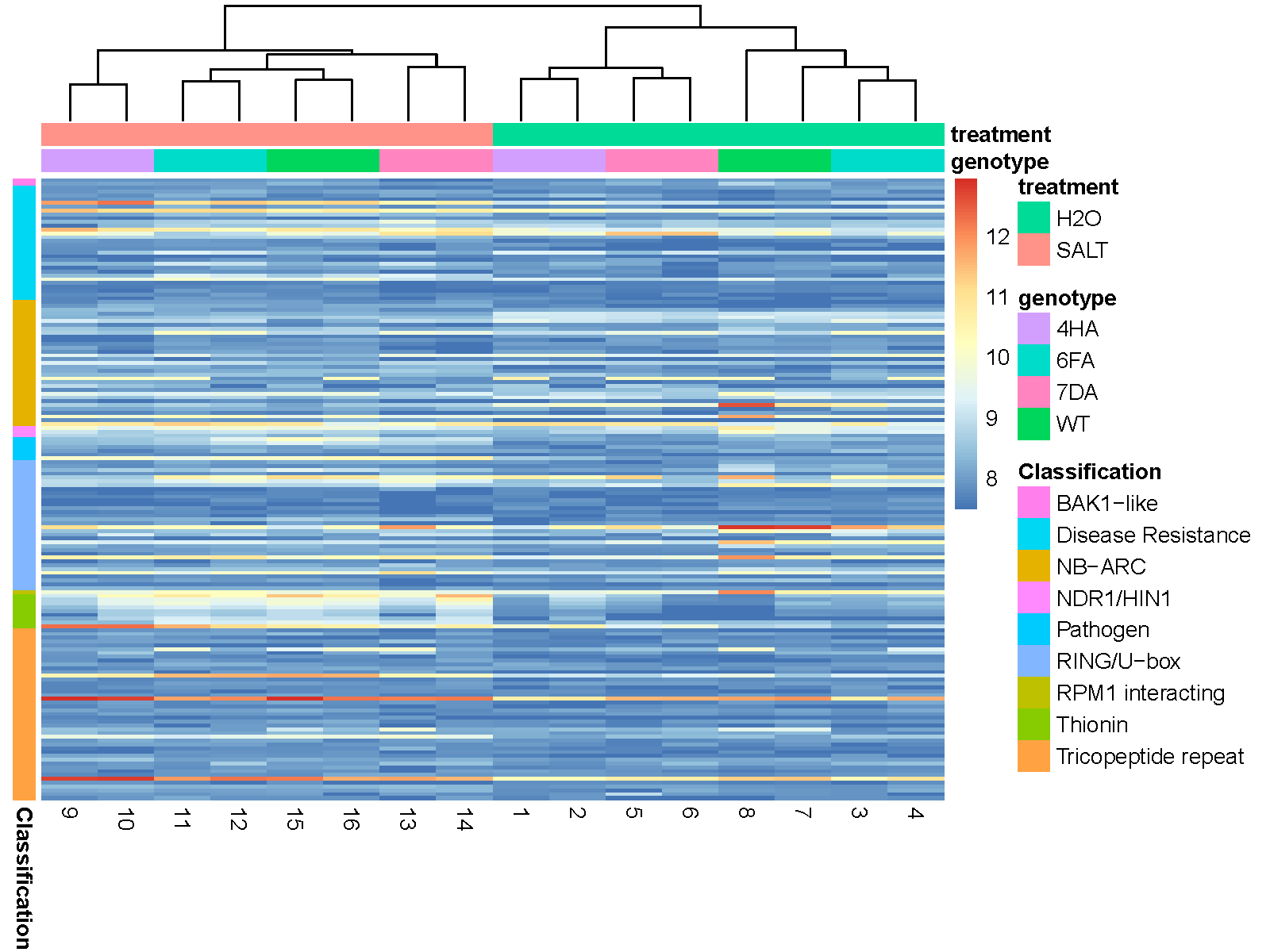

Supplement: Supplementary file 4 — Additional file 4: Figure S2. Heatmap of the 163 genes that are classified as disease resistance related genes that also pass the differential expression filter of p-value 0.05, FDR 0.5, and log2 fold changes less than − 2 or greater than 2. Values were transformed with variance stabilizing transformation. Treatments of H2O and salt, and genotypes of WT or transgenics 4HA, 6FA, 7DA are shown as colored bars above the columns. Classifications of disease related gene types are shown under the ‘Classification’ legend heading and are shown to the left of the rows. Samples (columns) are clustered using Euclidean distance and a dendogram is shown. [file 12870_2020_2341_MOESM4_ESM.png]

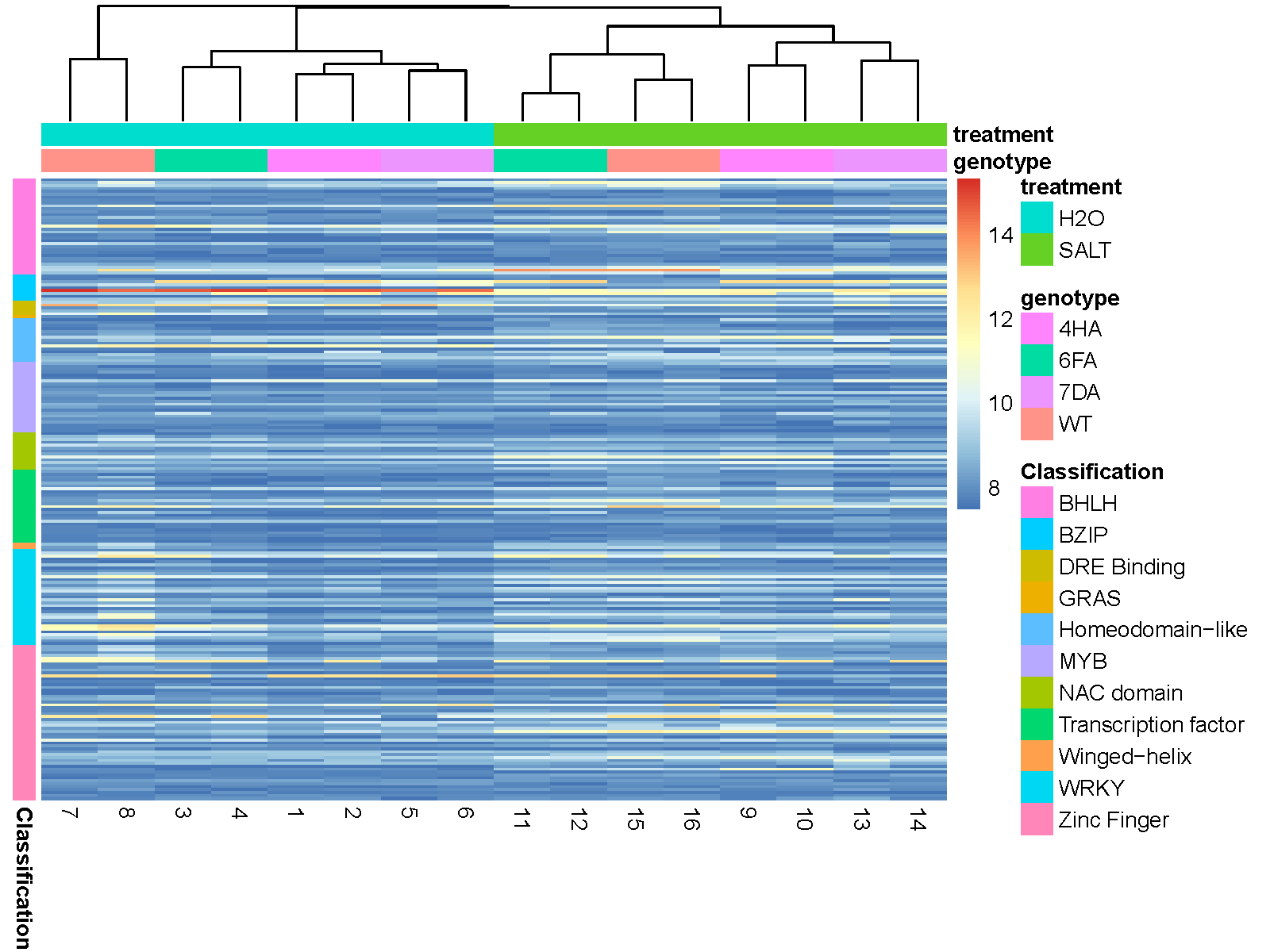

Supplement: Supplementary file 5 — Additional file 5: Figure S3. Heatmap of the 213 genes that are classified as transcription factors that also pass the differential expression filter of p-value 0.05, FDR 0.5, and log2 fold changes less than − 2 or greater than 2. Values were transformed with variance stabilizing transformation. Treatments of H2O and salt, and genotypes of WT or transgenics 4HA, 6FA, 7DA are shown as colored bars above the columns. Gene classifications of transcription factor types are shown under the ‘Classification’ legend heading and are shown to the left of the rows. Samples (columns) are clustered using Euclidean distance and a dendogram is shown. [file 12870_2020_2341_MOESM5_ESM.png]

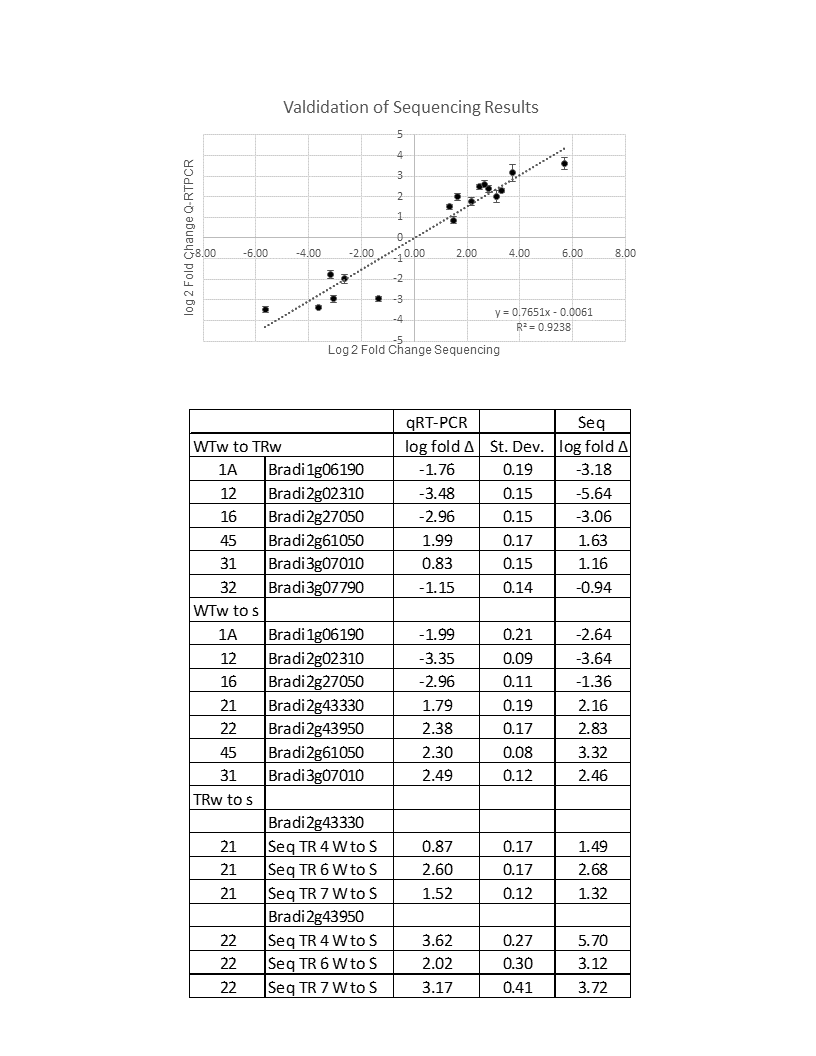

Supplement: Supplementary file 7 — Additional file 7: Figure S4. Quantitative RT-PCR validation of RNA-Seq data. Log2 fold change values obtained by qRT-PCR are plotted against the RNA-Seq data. Primers used for qRT-PCR are listed in Supplementary Table 1. [file 12870_2020_2341_MOESM7_ESM.png]
